# The pro-invasive factor COL6A2 serves as a novel prognostic marker of glioma

**DOI:** 10.3389/fonc.2022.897042

**Published:** 2022-11-25

**Authors:** Jinchao Zhu, Qingyuan Lin, Haiyan Zheng, Yamin Rao, Tianhai Ji

**Affiliations:** Department of Pathology, Shanghai Ninth People’s Hospital, Shanghai Jiao Tong University School of Medicine, Shanghai, China

**Keywords:** COL6A2, glioma, prognosis, tumor-infiltrating immune cells, immunomodulatory genes

## Abstract

**Background:**

Glioma is an incurable malignant lesion with poor outcome characterized by easy recurrence after surgery with or without radiotherapy and chemotherapy. Studies have shown that *COL6A2* is closely related to the tumorigenesis and development of a variety of tumors. However, the role of *COL6A2* in glioma and the relationship between *COL6A2* and tumor infiltrating immune cells remain unclear.

**Methods:**

Western blot, real-time *PCR*, a tissue microarray and immunohistochemistry were applied to detect *COL6A2 mRNA* and protein amounts in glioma, and all experiments were repeated three times. A tissue microarray of glioma samples was used for prognostic analysis. Detection of *COL6A2* co-expression with immune genes using immunohistochemical methods, and tumor modeling using nude mice for prevention and treatment studies. Based on the mRNA expression of *COL6A2*, patients with glioma in *TCGA* were divided into the low and high *COL6A2* expression groups, and *GO* and *KEGG* pathway analyses were performed. A *PPI* network was constructed using STRING, and the associations of *COL6A2* with tumor-infiltrating immune cells and immune genes were analyzed in the *CIBERSORT* and *TISIDB* databases. *COL6A2* mRNA and protein amounts were increased in glioma.

**Results:**

Multiple-database and tissue microarray analyses showed that *COL6A2* expression in glioma was associated with poor prognosis, Tissue microarray showed that *COL6A2* was the highest expressed in *WHO* IV and significantly higher in *TCGA-GBM* than in *TCGA-LGG*. Immunohistochemistry can well demonstrate the co-expression of COL6A2 with immune genes in a tumor model established in nude mice, showing that interference with *COL6A2* expression may have an inhibitory effect on tumors. The mRNA expression of *COL6A2* was involved in 22 *KEGG* pathways, and *GSEA* analysis showed that 28 and 57 gene sets were significantly enriched at nominal p values <0.01 and <0.05, respectively, protein network revealed a tight interaction between *COL6A2* and *SPARC.* The *CIBERSORT* database indicated that *COL6A2* was correlated with 15 types of tumor-infiltrating immune cells, including M2 macrophages, CD8 T cells, neutrophils, gamma delta T cells, activated CD4 memory T cells, follicular helper T cells, M0 macrophages, M1 macrophages, regulatory T cells (Tregs), activated NK cells, eosinophils, activated mast cells, monocytes, activated dendritic cells, and resting CD4 memory T cells. The *TISIDB* database indicated that *COL6A2* was significantly correlated with lymphocytes such as regulatory T cell, Type 17 T helper cell, Type 1 T helper cell, and immunomodulatory genes. In addition, *COL6A2*-related immune regulatory genes show that most immune regulatorygenes have prognostic value for glioma, and high-risk immune genes are notconducive to the survival of glioma patients.

**Conclusions:**

*COL6A2*-related immune regulatory genes show that most immune regulatory genes have prognostic value for glioma, and high-risk immune genes are not conducive to the survival of glioma patients. *COL6A2* may be a novel potential prognostic biomarker of glioma and associated with tumor-infiltrating immune cells in the tumor microenvironment, and interference with *COL6A2* expression can inhibit tumor growth, which suggests *COL6A2* as a potential target for future treatment.

## Introduction

Glioma is a common highly malignant brain tumor with unclear boundary, infiltrative growth and frequent recurrence. At present, surgery combined with radiotherapy and chemotherapy is the most effective treatment, even though the tumor always recurs after those treatments (1). Based on Yan’s report, the five-year survival rate of patients with glioma was only 9.8% (2), including only one-year survival data for grade III and IV glioma (3). It is a great challenge to develop a more effective treatment.

Collagen VI –α2 (COL6A2) is located in zone 2 of the long arm of chromosome 21 (4), contributing to COL6 protein synthesis. Farhan HAQ et al. carried out detailed sequence, phylogenetic and homology analyses of 44 collagen genes to examine the complexity of the collagen gene family, and revealed the diversity of the collagen gene family in sequence, structure and function (5). Mutation of this gene is associated with Bethlem myopathy and Ullrich sclerosing muscular dystrophy as well as congenital atrial septal defect. It was reported by Gittenberger et al. that COL6A2 expression is increased in congenital atrial septal defect (6). Weston et al. found that COL6A2 gene expression is negatively regulated by vascular endothelial growth factor A (VEGF-A), indicating inhibited VEGF-A gene expression may upregulate COL6A2 (7). Meanwhile, COL6A2 can promote the proliferation of skeletal muscle cells, and reduced COL6A2 gene expression reduces the proliferation of skeletal muscle cells, leading to skeletal muscle dysfunction and congenital muscle atrophy (8).

Recently, it was reported that collagen is closely associated with the development of tumors. In a study, collagen expression was associated with poor prognosis in gastric cancer (9). In addition, COL6A2 is associated with low OS in patients with high-grade serous ovarian cancer, and overexpressed in mammary cancer (10). COL6A2 participates in the development and progression of bladder cancer (11), and plays a role in MAPK and Akt signaling by increasing p38 MAPK phosphorylation and reducing AKT phosphorylation (12). Therefore, COL6A2 may participate in the course of lesion development.

Since the specific mechanism of COL6A2 in glioma is unclear, the purpose of this study was to comprehensively analyze the expression and significance of COL6A2 in glioma through the TCGA, CGGA and GEO databases, to verify COL6A2 expression by WB, PCR and immunohistochemistry and to determine the association of COL6A2 expression with prognosis in glioma.

## Methods

### Glioma dataset source and preprocessing

We obtained the gene-expression data and complete clinical annotation from the Gene-Expression Omnibus (GEO, http://www.ncbi.nlm.nih.gov/geo/), Chinese Glioma Genome Atlas (CGGA, Home | CGGA - Chinese Glioma Genome Atlas) and Cancer Genome Atlas (TCGA, GDC (cancer.gov)) databases. Patients without survival information were excluded. We selected a qualified glioma cohort (GSE4412) from GEO, mRNAseq_693 and mRNAseq_325 datasets downloaded from CGGA and TCGA-glioma (including TCGA-GBM and TCGA-LGG) were analyzed in this study.

### Western blot

For Western blot, the main instruments used included a horizontal shaker (Micrococl 17, Jiangsu Haimenqi Linbei Instrument Manufacturing Co., Ltd., BiOCl 17), a microplate reader (BioTek, Epoch 2) and an electronic balance (CPA). The main materials were RIPA lysis buffer (Biyuntian, p0013b), BCA protein concentration determination kit (Biyuntian, p0010) and PBS (Symantec). Antibodies were purchased from Abcam, and used at 1:5000. After the cells were cultured in 6-well plates, 150µl of lysis buffer was added to each well, and protein concentration in the lysate was quantified with the BCA kit. The cell lysates were loaded onto SDA-PAGE gels and separated by electrophoresis. The protein bands were transferred onto PVDF membranes, and the target molecule was detected by Western blot. The experiment was conducted in triplicate and repeated three times.

### Real-time quantitative PCR

RNA was extracted from glioma cells with TRIzol reagent and quantitated on a spectrophotometer. Then, the miRNA cDNA strand kit (AT341, TransGen Biotechnology), SG Fast qPCR Master Mix (b639273, S-ANGON) and Real-time fluorescence quantitative PCR instrument were used for qPCR. The first strand of RNA cDNA was synthesized with TransScript All-in-One SuperMix, with gDNA removal. The first strand of miRNA cDNA was synthesized by adding transcript miRNA RT enzyme mix, TS miRNA reaction mix and ddH2O reagen to nucleus-free PCR tubes, for fluorescence quantitative PCR detection. The following primer sets were used for RT-PCR: COL6A2-F, TACGGAGAGTGCTACAAGGTG, COL6A2-R, GGTCCTGGGAATCCAATGGG, GAPDH-F, GGAGCGAGATCCCTCCAAAAT, GAPDH-R, GGCTGTTGTCATACTTCTCATGG.

### Tissue microarray and immunohistochemistry

The study protocol was approved by the ethics committee of the General Hospital of Central War Zone and The Ninth People’s Hospital, Shanghai Jiaotong University School of Medicine. The glioma tissue microarray was provided by the abovementioned hospitals. Anti-COL6A2 antibodies were purchased from Abcam and used at 1:250. Tissue samples were obtained from the Ninth People’s Hospital Affiliated to Shanghai Jiaotong University. The tissue samples were sectioned, dewaxed and incubated with primary and secondary antibodies, and then observed under a microscope after dehydration and sealing. All sections were scored by two independent pathologists in a blinded fashion. Specifically, the intensity of positive staining in the cytoplasm was scored on a scale of 0-3 (0 shows negative, 1 shows light brown, 2 shows medium brown, 3 shows dark brown). The percentage of positively stained cells was scored as 1 (1-25%), 2 (26-50%), 3 (51-75%) or 4 (76-100%). When duplicated cores showed different staining, the higher score from the two tissue cores was considered the immune reactivity score (IRS). COL6A2 staining patterns were defined as low (IRS, 0-7) and high (IRS, 8-12). COL6A2 was co-expressed with immune genes at concentrations of 1:250, 1:500.

### Experimental modeling of nude mouse tumors

The nude mouse is an animal model developed in the last decade or so with the development of modern medicine, especially the needs of oncology and immunology research. Due to the immunodeficiency of the nude mouse, it does not reject tissue transplants from xenografts under certain circumstances, and therefore, it can be used to establish an ideal tumor model for human tumor transplantable experimental animals. BALB/c nude mice were purchased from Shanghai Jihui Biotechnology Co. Thirty BALB/c nude mice aged 5-8 weeks and weighing about 18-20 g were selected and grouped as follows, U87-Ctrl group and U87-shRNA group. Tumor cells of the logarithmic growth phase overexpression stable transfer strain and its control stable transfer strain were collected, blown uniformly and adjusted to a cell concentration of 2 × 108 cells/ml, respectively, in an inoculum volume of 5 ul. The microsampler containing the cell lines was slowly lowered to a depth of 2.5 mm below the skull. The cell line is slowly injected into the brain tissue *via* a micro-constant flow pump. The rate was approximately 1ul/2min. 3 weeks after cell inoculation, imaging was performed with an IVIS Lumina XR (company, PerkinElmer) small animal imager. After isoflurane anesthesia, tumor-bearing mice are placed in the imaging system cassette in order of tumor size from largest to smallest, and used to detect tumor cell signals.

### Kaplan-Meier survival analysis

The Kaplan-Meier database was used to determine the overall survival (OS) rate of glioma patients, as a standard processing pipeline that analyzes 698 tumor and 5 normal TCGA-glioma specimens, there were data of 1108 clinical samples from TCGA-glioma and 85 tumor samples from GSE4412. Finally, mRNAseq_325. RSEM-genes and mRNAseq_693.RSEM-genes datasets and the corresponding clinical data were downloaded from CGGA. Survival curves for differential COL6A2 expression were analyzed with the “survival package” to determine the correlation of gene expression with glioma patient prognosis.

### Independent prognostic analysis and ROC curve plotting

To evaluate the associations of survival or prognosis with clinicopathological factors and risk score, univariate and multivariate Cox regression analyses were performed with the Survival R package. The survival ROC R software package was used to generate receiver operating characteristic (ROC) curves to assess the values of different clinicopathological factors and the risk score in predicting survival time.

### Co-expression network

The limma package was utilized to process the TCGA-glioma data set, with a logFC threshold of 2. The output co-expressed genes, Nodes and edges files were exported, and visualization was performed with Cytoscape 3.6.1. Gene Ontology (GO) and KEGG pathway analyses were carried out.

### Gauging the immune response of 22 TIICs (Tumor-Infiltrating Immune Cells) in glioma *via* CIBERSORT and TISIDB

CIBERSORT (https://cibersort.stanford.edu/) (13), a deconvolution algorithm based on gene expression, can evaluate the expression changes of a group of genes relative to all other genes in the sample. Therefore, TIIC levels can be accurately estimated using this process. The TISIDB database was used to analyze the associations of COL6A2 with the infiltration of immune cells (B cells, CD4+T cells and CD8+T cells) in gliomas (GBM in gliomas was selected). TISIDB (http://cis.hku.hk/TISIDB/index.php) allows users to determine the roles of specific genes in tumor immune interactions through high-throughput data analysis.

### Construction and functional analysis of a PPI network of COL6A2-related immunomodulatory genes

The interactions of immune regulatory genes were analyzed in the string database. In addition, Gene Ontology (GO) and KEGG pathway analyses of immunomodulatory genes were performed in the WebGestalt database (www.webgestalt.org).

### Development of a COL6A2-related immunomodulatory prognostic signature

The expression levels of 32 immunomodulatory genes were extracted and analyzed in gliomas, and univariate Cox regression analysis was used for the primary screening of survival-related genes. To prevent omissions, 0.05 was set as the cutoff p-value, and 28 survival-related genes were identified for further analysis. A multivariate COX prognostic evaluation model was built, according to the median risk score, TCGA-glioma patients were divided into low-risk and high-risk groups. The R “survival” package was used to evaluate the significance of OS difference between the high- and low-risk groups. Furthermore, according to the median risk score, univariate and multivariate Cox independent prognostic analyses of clinical characteristics were performed. The survival ROC R package was used to generate a time-dependent receiver operating characteristic (ROC) curve for assessing the values of different clinicopathological factors and the risk score in survival time prediction.

### Construction and validation of a nomogram

After univariate Cox regression analysis, all significant prognostic factors were screened by multivariate Cox regression analysis to construct a predictive nomogram, and the rms R software package was used to evaluate the odds of 1-, 2-, and 3-year OS in TCGA-glioma patients. The C index and AUC were utilized to quantitatively evaluate the discriminative performance of the nomogram (14). Calibration charts were also used to graphically evaluate the discriminability of the nomogram (15).

### Statistical analysis

Western blot bar charts were generated with the GraphPad Prism 5 software. The R Statistical software (version 4.0.3) was used for data analysis. Survival analysis used the log-rank test and the Kaplan-Meier method. To assess the independent prognostic values of risk models, univariate and multivariate Cox regression models were utilized.

## Results

### COL6A2 expression in human glioma

**Figure 1:**
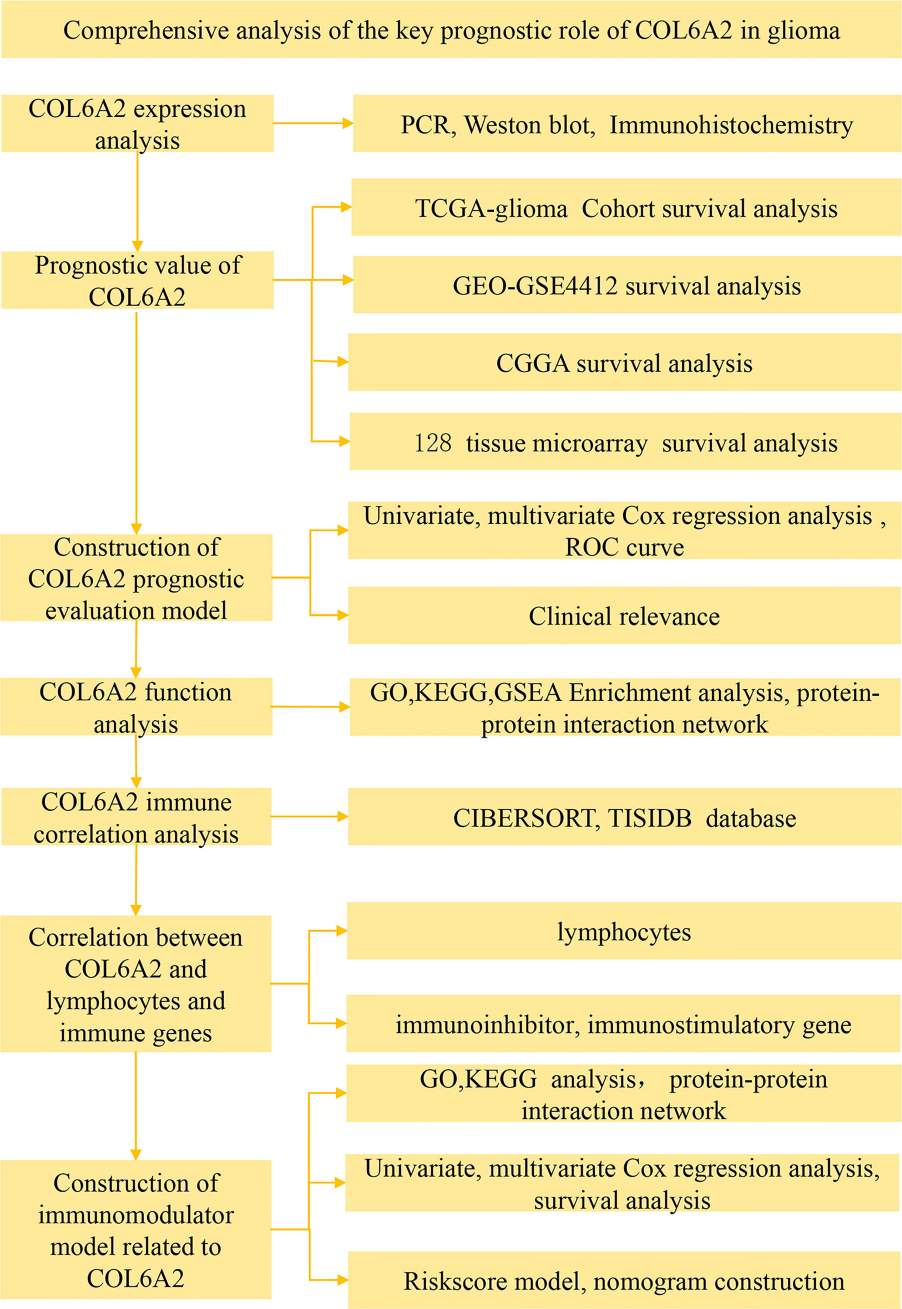
Flow chart of data collection and analysis COL6A2 expression in glioma.

**Figure 2:**
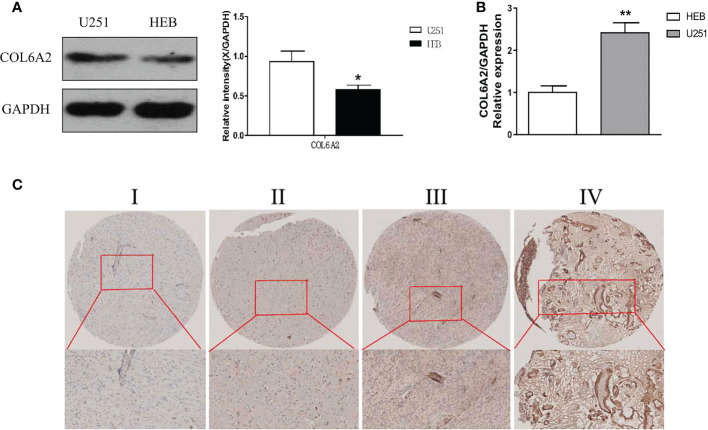
Expression of COL6A2 in human glioma cells. **(A)** The protein levels of COL6A2 in human glioma cells. **(B)** The mRNA levels of COL6A2 in glioma cells as shown by qPCR analyses (*p<0.05, **p<0.01). **(C)** IHC assay determined COL6A2 expression in different WHO grades glioma tissues. I: WHO I, II: WHO II, III: WHO III, IV: WHO IV.

Our data collection and analysis as shown on **Figure 1** to identify key biomarkers in glioma, we performed multicenter screening and validation, and finally identified the key marker COL6A2 (**Table S1**). Compared with LGG, COL6A2 has a significant difference in GBM expression in the TCGA database (**Figure S1A**). Western blot was performed to examine COL6A2 protein levels in glioma U251 and normal HEB cells. The expression of COL6A2 was increased in U251 cells compared with HEB cells (**Figure 2A**), The CCLE dataset shows that COL6A2 is expressed in multiple cell lines (**Figure S1B**, **Table S2**). Real-time PCR was performed to assess the mRNA expression of COL6A2 in glioma cells. The results showed significantly increased COL6A2 mRNA amounts in U251 cells (**Figure 2B**). Moreover, COL6A2 was detected in glioma tissues by immunohistochemistry and Significant differences in WHO IV expression (**Figure 2C**; **Figure S1C**, **Table 1**). These findings suggested that the high expression of COL6A2 in glioma may affect the biological function of glioma.

**Table 1:** Tissue microarray clinical features.

		COL6A2 expression		
Clinicopathological feature	[ALL] N=79	0 N=39	1 N=40	p.overall
gender	0.62 (0.49)	0.64 (0.49)	0.60 (0.50)	0.711
age:				<0.001
<=41	31 (39.2%)	26 (66.7%)	5 (12.5%)	
>41	48 (60.8%)	13 (33.3%)	35 (87.5%)	
grade:				<0.001
I	4 (5.06%)	4 (10.3%)	0 (0.00%)	
II	34 (43.0%)	24 (61.5%)	10 (25.0%)	
III	19 (24.1%)	6 (15.4%)	13 (32.5%)	
IV	22 (27.8%)	5 (12.8%)	17 (42.5%)	

### Tumor imaging *in vivo* in mice

Thirty BALB/c nude mice were divided into two groups, the U87-Ctrl group and the U87-shRNA group. After 3 weeks of cell inoculation, we performed mouse fluorescence imaging assays on both groups and found that interference with COL6A2 expression significantly inhibited tumor growth in mice compared with the control group, indicating that COL6A2 is a pro-oncogene in gliomas and high expression is detrimental to patient survival (**Figure S3A**).

### Expression patterns and prognostic significance of COL6A2 in glioma tissue microarray, TCGA, GEO and CGGA databases

**Figure 3:**
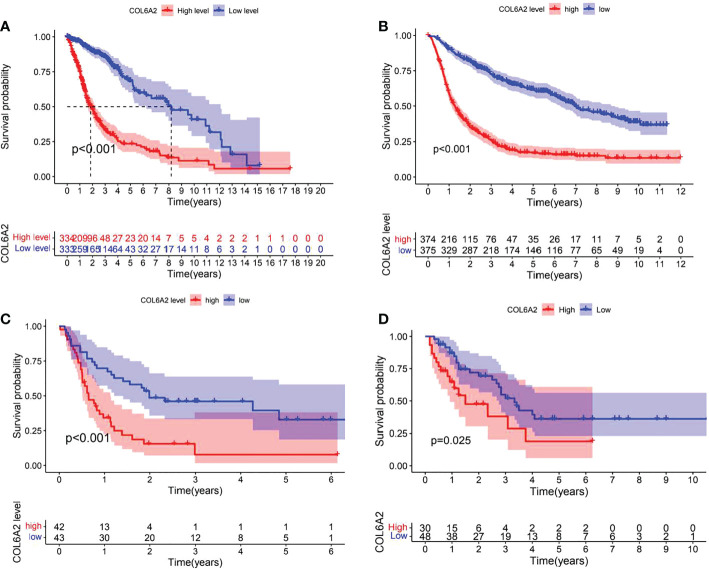
Effect of COL6A2 expression on prognosis of glioma. **(A)** Data indicate that the expression levels of COL6A2 are associated with poor prognostic survival in glioma patients in the TCGA cohort. **(B)** In CGGA database, high expression of COL6A2 is a poor prognostic factor for glioma patients. **(C)** In GEO database, high expression of COL6A2 is associated with poor prognosis of glioma patients. **(D)** In the follow-up data of 32 patients with glioma, the high expression of COL6A2 played an adverse role in glioma.

**Figure 4:**
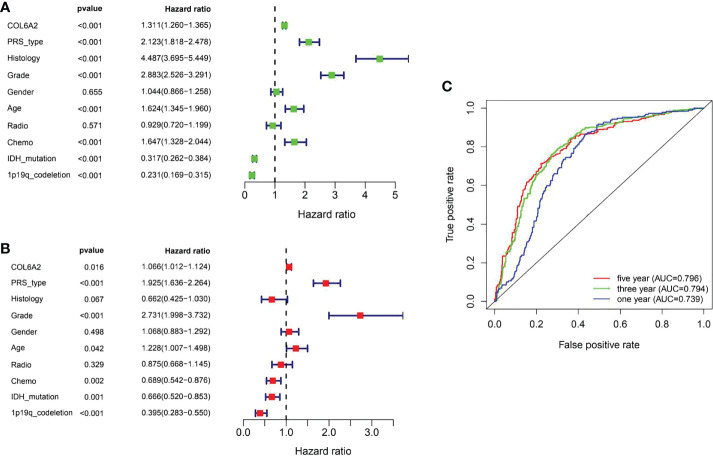
Evaluation of COL6A2 expression on the prognostic risk model of glioma patients in the CGGA. **(A)** Univariate analysis. **(B)** Multivariate Cox analyses. **(C)** The predictive accuracy of risk signature, five-year AUC=0.796, three year AUC=0.794, one year AUC=0.739.

**Figure 5:**
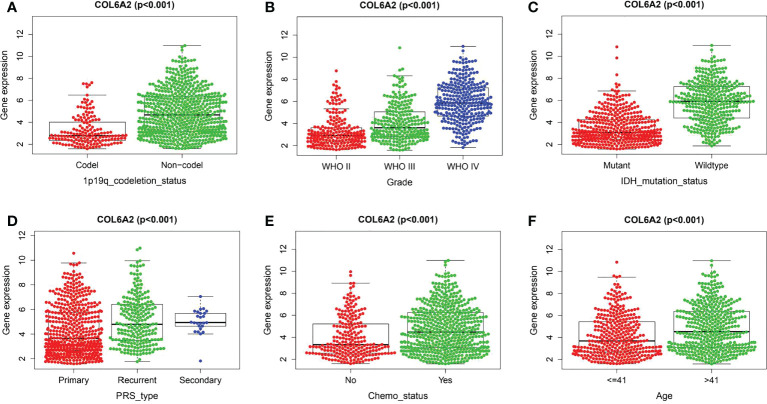
Correlation between COL6A2 expression and clinical traits in CGGA. **(A)** the expression of COL6A2 has a significant correlation with 1p19q codeletion. **(B)** In WHO grade, COL6A2 expression was higher with the higher grade. **(C)** COL6A2 expression was significantly correlated with IDH mutation, and the expression was high in the wild type. **(D)** The expression of COL6A2 is related to disease status, and the expression is the highest in secondary. **(E)** The expression of COL6A2 increased under chemotherapy. **(F)** COL6A2 was significantly correlated with age.

Kaplan-Meier curve analysis suggested that high COL6A2 expression was significantly associated with poor overall survival in the TCGA (p<0.001, **Figure 3A**), CGGA (p<0.001, **Figure 3B**) and GSE4412 (p<0.001, **Figure 3C**) datasets. Considering the important function of COL6A2, its expression levels were examined in various stages of glioma. A tissue microarray with samples from 128 glioma patients was constructed for this purpose. Patients were divided into two groups, including low- (IRS ≤ 7) and high- (IRS ≥ 8) COL6A2 expression groups, and COL6A2 in glioma tissue microarray (TMA) was detected by IHC. The tissue microarray (TMA) prognostic analysis corroborated the TCGA, CGGA and GEO databases, high expression was unfavorable to survival in glioma patients, and 5-year overall survival was significantly shorter in glioma patients with high COL6A2 expression levels compared with counterparts with low COL6A2 expression levels (p=0.025, **Figure 3D**). In addition, in the CGGA dataset, univariate (**Figure 4A**) and multivariate (**Figure 4B**) COX regression analyses indicated COL6A2 was associated with poor survival and constituted an independent prognostic factor in each dataset. COL6A2 also showed associations with age, gender, IDH mutation, PRS (P, Primary, R, Recurrent, S, Secondary), WHO grade, chemotherapy, 1p19q co-deletion and histology (**Table 2**). In receiver operating characteristic (ROC) analysis of COL6A2, AUC values for 1-, 3-, and 5-year survival were 0.739, 0.794, and 0.796 in glioma, respectively (**Figure 4C**). Therefore, COL6A2 has high predictive significance for patients with glioma. These results suggested that COL6A2 may be a prognostic factor in glioma. In addition, the associations of COL6A2 with the clinical characteristics of glioma patients were examined, and COL6A2 was highly expressed in wild type IDH cases (p<0.001). In patients with recurrent gliomas, COL6A2 was also highly expressed. In WHO classification, the higher the grade, the higher the expression of COL6A2 (**Figure 5**). The expression of COL6A2 was significantly higher in glioblastoma compared with other types of glioma.

**Table 2:** Univariable and multivariable analyses for each clinical feature.

Clinical feature	Univariate analysis			Multivariate analysis		
	HR	95%CI	P value	HR	95%CI	P value
COL6A2	1.31	1.26-1.36	3.13E-40	1.06	1.01-1.12	0.015
PRS_type	2.12	1.81-2.47	1.79E-21	1.92	1.63-2.26	2.60E-15
Histology	4.48	3.69-5.44	7.38E-52	0.66	0.42-1.02	0.067
Grade	2.8	2.52-3.29	1.44E-55	2.73	1.99-3.73	2.89E-10
Gender	1.04	0.86-1.25	0.65	1.06	0.88-1.29	0.498
Radio	0.92	0.71-1.19	0.57	0.87	0.66-1.14	0.329
Chemo	1.64	1.32-2.04	5.71E-06	0.68	0.54-0.87	0.002
IDH_mutation	0.31	0.26-0.38	3.84E-32	0.66	0.52-0.85	0.001
1p19q_codeletion	0.23	0.16-0.31	2.08E-20	0.39	0.28-0.54	3.95E-08

### GO and KEGG pathway analyses, and construction of a co-expression network

**Figure 6:**
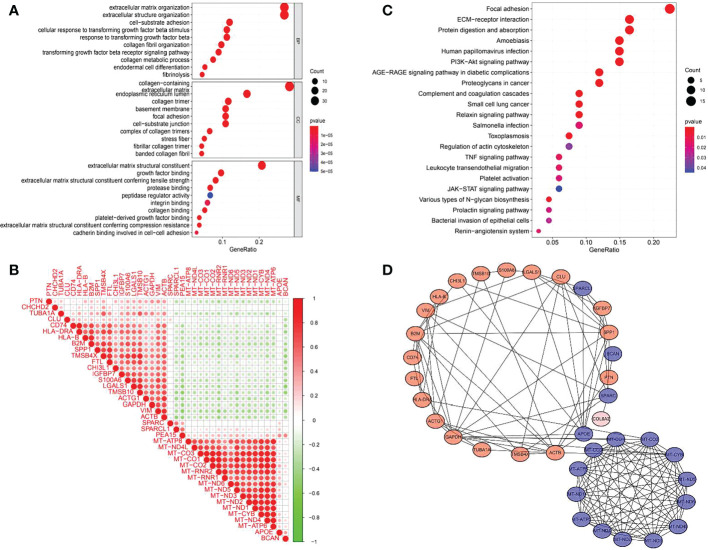
GO, KEGG enrichment analysis and gene co-expression of COL6A2. **(A)** GO enrichment analysis showed that the expression of COL6A2 was related to biological functions such as extracellular matrix organization, collagen-containing, and extracellular matrix structural constituent. **(B)** KEGG enrichment analysis showed that the expression of COL6A2 was related to the pathway functions such as Focal adhesion, ECM-receptor interaction, and Human papillomavirus infection. **(C)** Co-expression heat map of COL6A2 related genes, red, positive correlation, green, negative correlation. **(D)** Protein interaction network, red, upregulation, blue, down-regulation.

GO (gene ontology) is a database established by the Gene Ontology Consortium, which contains biological processes, cellular components and molecular functions. The top 10 GO enriched terms are shown in **Figure 6A**. The biological processes included extracellular matrix organization, extracellular structure organization, cell−substrate adhesion, cellular response to transforming growth factor beta stimulus, response to transforming growth factor beta, collagen fibril organization, transforming growth factor beta receptor signaling pathway, collagen metabolic process, endodermal cell differentiation and fibrinolysis. The GO enriched cellular components were collagen−containing extracellular matrix, endoplasmic reticulum lumen, collagen trimer, basement membrane, focal adhesion, cell−substrate junction, complex of collagen trimers, stress fiber, fibrillar collagen trimer and banded collagen fibril. The GO enriched molecular functions included extracellular matrix structural constituent, growth factor binding, extracellular matrix structural constituent conferring tensile strength, protease binding, peptidase regulator activity, integrin binding, collagen binding, platelet−derived growth factor binding, extracellular matrix structural constituent conferring compression resistance and cadherin binding involved in cell−cell adhesion. There were 22 KEGG pathways involved in COL6A2 expression (**Table 2**, **Figure 6B**). We used the “limma” R package to analyze gene expression differences in the TCGA-glioma dataset. The filtering threshold of log fold change was 2, and the adjusted p value threshold was 0.001. As a result, 40 differentially expressed genes were detected. The co-expressed genes (**Figure 6C**) were utilized to construct protein interaction networks (**Figure 6D**).

### GSEA enrichment analysis in the low- and high-COL6A2 expression groups

**Figure 7:**
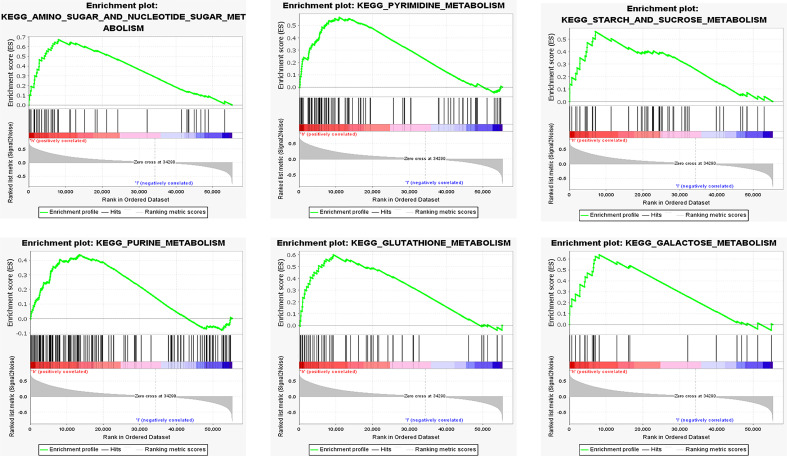
Functional enrichment analysis based on the risk model of the COL6A2 expressed by GSEA, KEGG pathways and oncogenic signatures in the high-risk group.

Enrichment analysis was performed of 351 samples with high COL6A2 expression. There were 97 gene sets with significant enrichment at FDR < 25%, 28 and 57 gene sets were significantly enriched at nominal p values < 0.01 and < 0.05, respectively. Six significantly upregulated hallmark gene sets involved in metabolism were selected, including “AMINO_SUGAR_AND_NUCLEOTIDE_SUGAR_METABOLISM”, “PYRIMIDINE_METABOLISM”, “STARCH_AND_SUCROSE_METABOLISM”, “PURINE_METABOLISM”, “GLUTATHIONE_METABOLISM”, and “GALACTOSE_METABOLISM”. The six gene sets are shown in **Table 3**, and enrichment analysis results are shown in **Figure 7**.

**Table 3:** KEGG pathway involved in glioma based on the expression level of COL6A2.

ID	Description	GeneRatio	BgRatio	P value	p.adjust
hsa04510	Focal adhesion	15/67	201/8108	5.42E-11	7.16E-09
hsa04512	ECM-receptor interaction	11/67	88/8108	1.02E-10	7.16E-09
hsa04974	Protein digestion and absorption	11/67	103/8108	5.75E-10	2.70E-08
hsa05146	Amoebiasis	10/67	102/8108	8.68E-09	3.06E-07
hsa04933	AGE-RAGE signaling pathway in diabetic complications	8/67	100/8108	1.45E-06	4.08E-05
hsa04610	Complement and coagulation cascades	6/67	85/8108	6.65E-05	0.001563
hsa05222	Small cell lung cancer	6/67	92/8108	0.000104	0.002087
hsa05205	Proteoglycans in cancer	8/67	205/8108	0.000264	0.004653
hsa05165	Human papillomavirus infection	10/67	331/8108	0.00035	0.005482
hsa04151	PI3K-Akt signaling pathway	10/67	354/8108	0.000595	0.008346
hsa04926	Relaxin signaling pathway	6/67	129/8108	0.000651	0.008346
hsa05145	Toxoplasmosis	5/67	112/8108	0.002248	0.026413
hsa00513	Various types of N-glycan biosynthesis	3/67	39/8108	0.003985	0.043227
hsa04668	TNF signaling pathway	4/67	112/8108	0.013573	0.134111
hsa04670	Leukocyte transendothelial migration	4/67	114/8108	0.014405	0.134111
hsa04614	Renin-angiotensin system	2/67	23/8108	0.015218	0.134111
hsa05132	Salmonella infection	6/67	249/8108	0.016516	0.136984
hsa04611	Platelet activation	4/67	124/8108	0.019052	0.147802
hsa04917	Prolactin signaling pathway	3/67	70/8108	0.019917	0.147802
hsa05100	Bacterial invasion of epithelial cells	3/67	77/8108	0.025555	0.180163
hsa04810	Regulation of actin cytoskeleton	5/67	218/8108	0.033709	0.226331
hsa04630	JAK-STAT signaling pathway	4/67	162/8108	0.044579	0.285712

### Composition of immune cells in the TCGA-glioma

**Figure 8:**
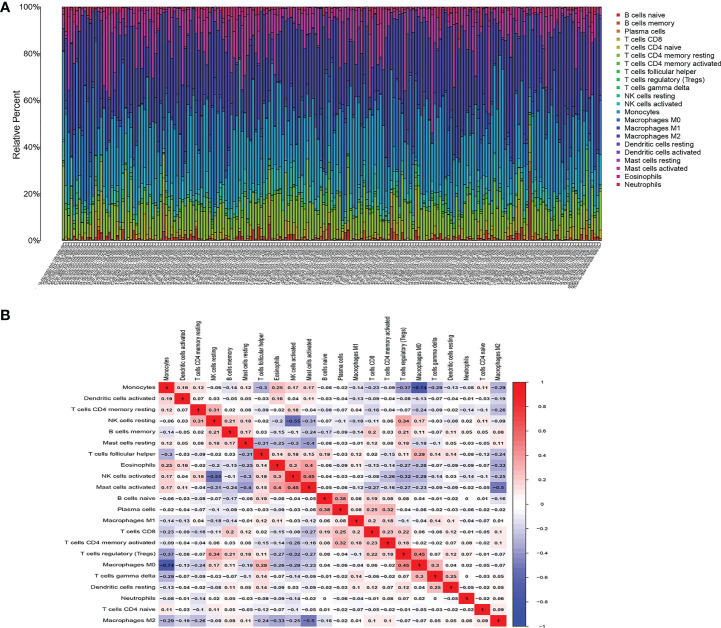
Composition of infiltrated immune cells between paired tumor and adjacent normal tissues in the TCGA cohort with CIBERSORT p < 0.05 for all eligible samples, 22 immune cells in TCGA cohort were filtered for analyzing, **(A)** Fractions of immune cells in 698 tumor and 5 normal samples in TCGA. **(B)** Correlation matrix of all 22 tumor-infiltrating immune cells proportions. Horizontal and vertical axes both represent tumor-infiltrating immune cells. tumor-infiltrating immune cells with higher, lower, and same correlation levels are shown in red, blue, and white, respectively.

We first studied the rates of infiltrated immune cells between paired tumor and normal tissues in the TCGA cohort, including 698 tumor and 5 normal samples, which were eligible with CIBERSORT p < 0.05. The expression matrix of 22 infiltrating immune cells is shown in **Figure 8A**. We found that M2 macrophages and monocytes were highly present in gliomas, and the correlations among the 22 TIICs were determined, ranging from weak to moderate. However, monocytes showed overt negative correlations with M0 macrophages, and M2 macrophages had significant negative correlations with activated mast cells **Figure 8B**.

### Relationship between COL6A2 expression and immune cell infiltration in glioma

**Figure 9:**
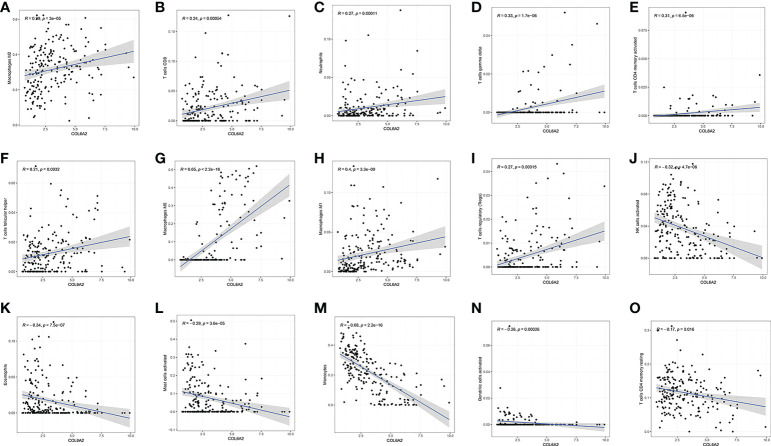
Correlation between COL6A2 expression and immune cells, **(A–I)** COL6A2 expression is positively correlated with immune cells such as Macrophages M2, T cells CD8, Neutrophils, T cells gamma delta, T cells CD4 memory activated, T cells follicular helper, Macrophages M0, Macrophages M1, and T cells regulatory (Tregs), **(J–O)** COL6A2 expression is negatively correlated with NK cells activated, Eosinophils, Mast cells activated, Monocytes, Dendritic cells activated, T cells CD4 memory resting.

The associations of COL6A2 expression with immune cells have not been reported so far. Therefore, we investigated whether COL6A2 expression was correlated with immune cell infiltration levels in glioma. Interestingly, COL6A2 expression was correlated with immune cell infiltration levels in glioma. The expression of COL6A2 was significantly positively correlated with the degrees of infiltration of M2 macrophages (*R* = 0.29, *p* = 2.8e−05), CD8+ T cells (*R* = 0.26, *p* = 0.00016), neutrophils (*R* = 0.26, *p* = 0.00013), gamma delta T cells (*R* = 0.24, *p* = 6e−04), activated CD4+ memory T cells (*R* = 0.26, *p* = 0.00021), follicular helper T cells (*R* = 0.22, *p* = 0.0018), M0 macrophages (*R* = 0.65, *p* < 2.2e−16), M1 macrophages *R* = 0.41, *p* = 6.7e−10) and regulatory T cells (Tregs, *R* = 0.23, *p* = 0.00098) (**Figure 9A–I**). In addition, COL6A2 expression was negatively correlated with activated NK cells (*R* = - 0.34, *p* = 5.5e−07), eosinophils (*R* = - 0.35, *p* = 2e−07), activated mast cells (*R* = - 0.26, *p* = 0.00019), monocytes (*R* = - 0.64, *p* < 2.2e−16), activated dendritic cells (*R* = - 0.28, *p* = 4.7e−05) and resting CD4 memory T cells (*R* = - 0.2, *p* = 0.0033) (**Figure 9J–O**). TIMER2.0(http://timer.cistrome.org/) analysis of the relationship between COL6A2 copy number and immune infiltration. The relationship between immune infiltration and somatic CNV regarding the expression of COL6A2 in glioma, the results show that the copy number of COL6A2 has different degrees of infiltration relationship with T cell CD8+, T cell CD4+, DC, etc (**Figure S2**). These results strongly suggested that COL6A2 plays a specific role in immune infiltration in glioma. Furthermore, in the performed co-expression of COL6A2 with the immune gene CD4, it was shown that COL6A2 and CD4 were well co-expressed in glioma tissues at different concentrations (**Figure S3B**).

### Associations of COL6A2 with lymphocytes in glioma

**Figure 10:**
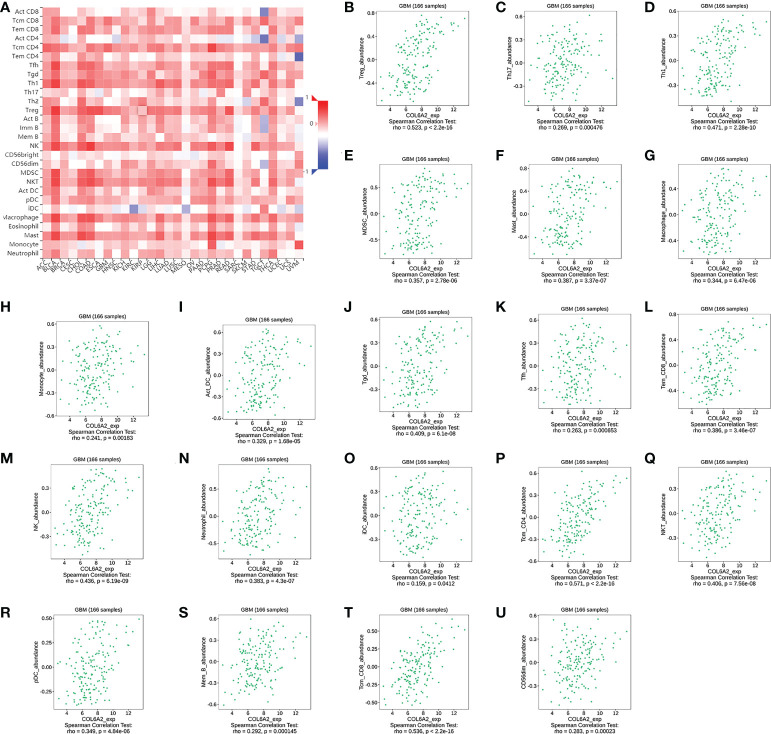
Correlation of COL6A2 expression with various B and T immune cell infiltration levels in GBM by TISIDB database. **(A)** Heatmap of the correlation between COL6A2 expression and lymphocytes, red, positive correlation, blue, negative correlation. **(B–U)** COL6A2 expression is significantly positively correlated with regulatory T cell, Type 17 T helper cell, Type 1 T helper cell lymphocytes in GBM. GBM, Glioblastoma.

Many studies have shown that the rate of lymphocytes infiltrating in tumors is an independent prognostic predictor in cancer (16, 17). Based on the above associations of COL6A2 expression with immune cells, the associations of COL6A2 expression with lymphocytes in GBM (glioblastoma multiforme) were further examined. The results showed that COL6A2 expression was positively correlated with regulatory T cell, type 17 T helper cell, type 1 T helper cell, myeloid derived suppressor cell (MDSC), mast cell, macrophage, monocyte, activated dendritic cell, gamma delta T cell (Tgd), T follicular helper cell (Tfh), effector memory CD8 T cell (Tem_CD8), natural killer cell, neutrophil, immature dendritic cell (iDC), central memory CD4 T cell (Tcm_CD4), natural killer T cell (NKT), plasmacytoid dendritic cell (pDC), memory B cell (Mem_B), central memory CD8 T cell (Tcm_CD8) and CD56dim natural killer cell (CD56dim) levels in GBM (**Figure 10A**). However, COL6A2 expression had no significant correlations with eosinophil, CD56bright natural killer cell (CD56bright), activated B cell (Act_B), immature B cell, type 2 T helper cell, effector memory CD4 T cell (Tem_CD4), activated CD8 T cell (Act_CD8) and activated CD4 T cell (Act_CD4) levels (**Figure 10A**). In addition, the expression of COL6A2 is positively correlated with regulatory T cells, type 17 T helper cells, type 1 T helper lymphocytes, etc. in glioblastoma (**Figure 10B–U**).

### COL6A2 expression is correlated with immunomodulators in glioma

**Figure 11:**
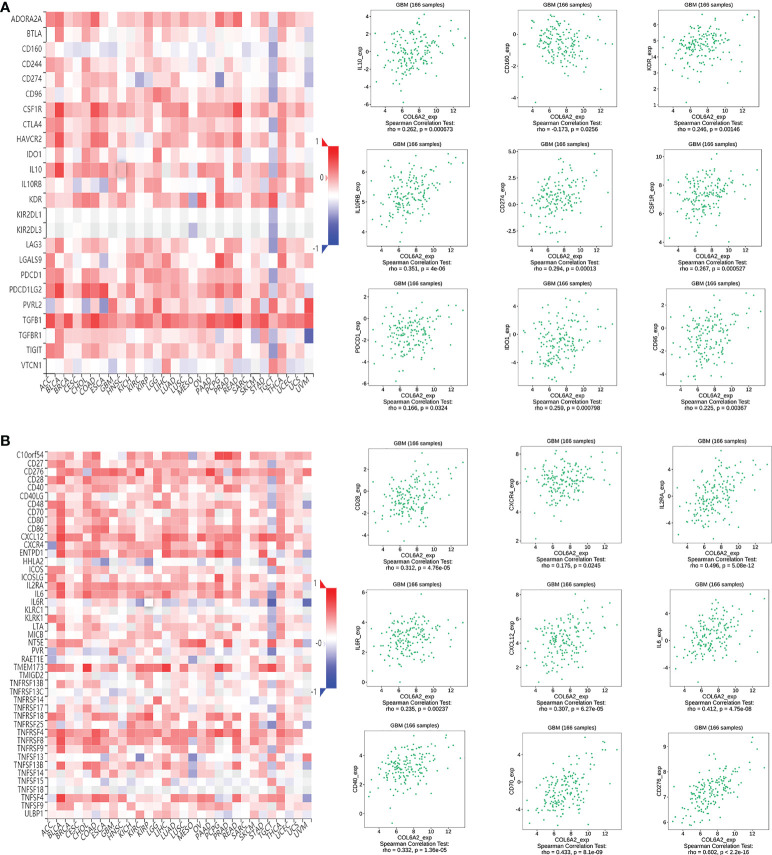
COL6A2 expression is associated to immunomodulatory genes. **(A)** Among immunoinhibitor genes, COL6A2 expression has both positive and negative correlations with IL10, CD160, KDR and other genes in GBM. **(B)** COL6A2 expression has both positive and negative correlations with CD28, CXCR4, IL2RA and other immunostimulatory genes in GBM. GBM, glioblastoma.

**Figure 12:**
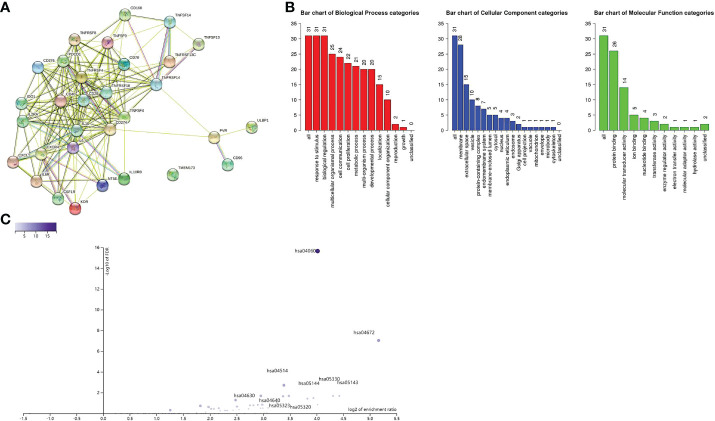
**(A)** Protein interaction between immune genes. **(B)** Immune genes are related to response to stimuli, membrane, protein binding and other functions. **(C)** The relationship between immune gene and enrichment pathway, and marked significant enrichment pathway.

Immunomodulators, including immunoinhibitory, immunostimulatory and MHC molecules. The associations of COL6A2 expression with immunomodulatory genes in glioma were examined. The results showed that among immunoinhibitory molecules, COL6A2 was mainly associated with CD96, CD160, CD274 CSF1R, IDO1, IL10, IL10RB, KDR and PDCD1 (**Figure 11A**), moreover, the expression of COL6A2 was also correlated with immunostimulatory molecules, including CD28, CD40, CD70, CD276, CXCL1, CXCR4, IL2RA, IL6, IL6R, NT5E, PVR, TMEM173, TNFRSF4, TNFRSF8, TNFRSF13C, TNFRSF14, TNFRSF18, TNFSF4, TNFSF9, TNFSF13, TNFSF14 and ULBP1 (**Figure 11B**). Protein interaction network (string database), GO enrichment and KEGG pathway analyses were performed for these 9 immunosuppressors and 22 immunostimulators. In the protein interaction network, all these genes interacted with each other except TMEM173 (**Figure 12A**). GO enrichment analysis showed that in BP, immunomodulator genes were mainly involved in response to stimulus, biological regulation and multicellular organic process (**Figure 12B**). In KEGG pathway analysis, we identified the most significant pathways for the immunomodulatory genes (**Figure 12C**).

### Identification of COL6A2-related immunostimulatory genes with significant prognostic value in glioma

**Figure 13:**
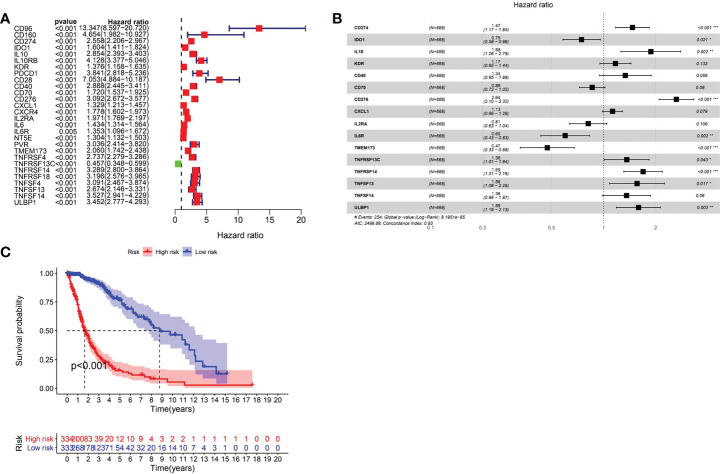
Prognostic evaluation of immune genes for glioma. **(A)** Related immune genes with significant prognosis in glioma. **(B)** Prognostic gene model construction. Shows that CD274, IL10, CD276, TNFRSF13C, TNFRSF14, TNFRSF13, ULBP1 are poor prognostic factors for patients with glioma, IDO1, IL6R, and TMEM173 are protective factors for glioma. **(C)** The prognostic survival analysis of immune genes for glioma showed that the high expression group is not conducive to the survival of glioma patients.

**Figure 14:**
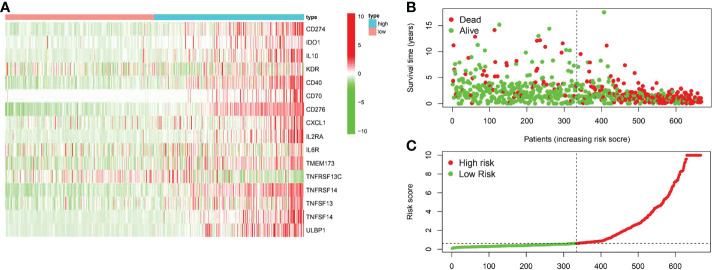
The prognostic value of the risk model of the 16 COL6A2 related immunostimulatory genes in the TCGA cohort. **(A)** The heatmap displayed the expression levels of COL6A2 related immunostimulatory genes in the high-risk and low-risk groups. **(B)** The scatterplot based on the survival status of each sample. The green and red dots represent survival and death, respectively. **(C)** The risk curve based on the risk score of each sample.

A total of 31 COL6A2 related immunostimulatory genes had significant expression. Among them, 28 COL6A2 genes were significantly related to survival rate in TCGA glioma patients (P < 0.01), including 1 low-risk immunostimulatory gene (hazard ratio (HR) < 1) and 27 high-risk immunostimulatory genes (HR > 1) (**Figure 13A**). Subsequently, multivariate Cox analysis further screened 16 immunostimulatory genes with prognostic significance from the above 27 COL6A2 related immunostimulatory genes, including 5 low-risk and 11 high-risk immunostimulatory genes (**Figure 13B**, **Table 3**). The 16 immunostimulatory genes included CD274, IDO1, IL10, KDR, CD40, CD70, CD276, CXCL1, IL2RA, IL6R, TMEM173, TNFRSF13C, TNFRSF14, TNFSF13, TNFSF14 and ULBP1 **Table 4**. According to the risk score formula and the calculated median risk score, patients with glioma were divided into the high-risk and low-risk groups. Kaplan-Meier survival analysis showed that overall survival (OS) was reduced in the high-risk group compared with the low-risk group (**Figure 13C**). The expression heatmap of these 16 COL6A2-related immunostimulatory genes in glioma patients showed that CD274, IDO1, IL10, KDR, CD40, CD70, CD276, CXCL1, IL2RA, IL6R, TMEM173, TNFRSF14, TNFSF13, TNFSF14 and ULBP1 were highly expressed in the high-risk group, while in the low-risk group, TNFRSF13C was up-regulated (**Figure 14A**), indicating that the risk score of glioma had a prognostic value. The risk curves and scatter plots were applied to describe the risk scores and related survival status of glioma patients. The results indicated that the occurrence of mortality depended on the risk score (**Figures 14B, C**). Therefore, the above findings suggested that 16 COL6A2-related immunostimulatory genes had prognostic significance in glioma.

**Table 4:** COL6A2 related prognostic immunomodulator gene.

id	coef	HR	95%CI	p value
CD274	0.38	1.46	1.17-1.83	0.000812
IDO1	-0.29	0.74	0.58-0.95	0.021051
IL10	0.62	1.87	1.26-2.79	0.001904
KDR	0.15	1.17	0.95-1.44	0.133457
CD40	0.29	1.33	0.94-1.88	0.099048
CD70	-0.15	0.85	0.72-1.01	0.080249
CD276	0.97	2.63	2.09-3.32	1.75E-16
CXCL1	0.11	1.12	0.98-1.28	0.079493
IL2RA	-0.2	0.81	0.63-1.04	0.106481
IL6R	-0.51	0.59	0.43-0.82	0.002001
TMEM173	-0.74	0.47	0.33-0.67	4.28E-05
TNFRSF13C	0.3	1.36	1-1.83	0.043017
TNFRSF14	0.52	1.6	1.3-2.19	5.96E-05
TNFSF13	0.44	1.56	1.08-2.25	0.016777
TNFSF14	0.3	1.35	0.98-1.86	0.059952
ULBP1	0.46	1.59	1.18-2.13	0.001903

### Immunostimulatory genes-related prognostic nomogram

**Figure 15:**
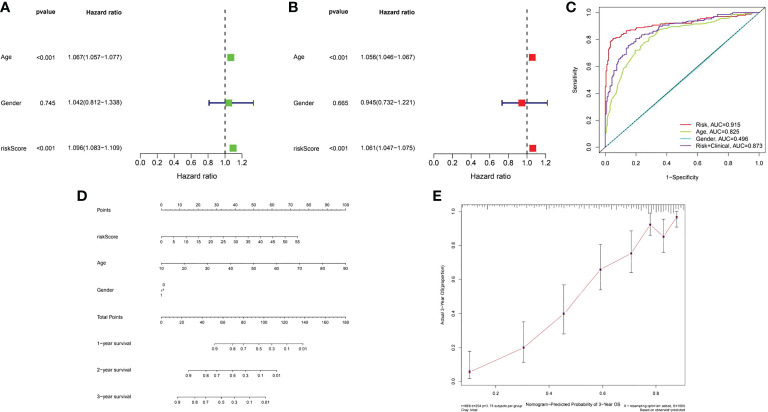
Assessment of the prognostic risk model of the 16 COL6A2 related immune genes in glioma. **(A)** The univariate and **(B)**, multivariate Cox regression analysis of risk model score and clinical features regarding prognostic value. **(C)** The AUC for risk model score and clinical features according to the ROC curves. Clinical features: age, gender, Risk, AUC=0.915, age, AUC=0.825, gender, AUC=0.496, risk + clinical, AUC=0.873. **(D)** Nomogram predicting OS of glioma patients from TCGA. **(E)** The calibration plot of the nomogram, the y‐axis represents actual survival, and the x‐axis represents nomogram predicted survival.

A risk score system for predicting the prognosis of glioma patients was developed. According to the median cut-off value of risk score, patients in each cohort were divided into the low-risk and high-risk groups. Univariate and multivariate Cox regression analyses of risk model score and clinical features showed that age was a poor prognostic factor of immunomodulator genes (**Figures 15A, B**). In addition, based on risk score, the values of age and gender in predicting survival in glioma patients were determined by receiver operating characteristic (ROC) curve analysis. The area under the curve (AUC) for the risk score was 0.915 in the TCGA-glioma cohort, versus 0.825 and 0.496 for age and gender, the AUC for risk score + clinical features was 0.873 in the TCGA-glioma. Based on the above multivariate Cox regression results, we developed a nomogram (**Figures 15C–E**) to predict 1, 2 and 3 year OS. These results suggested that the 16 COL6A2-related immunostimulatory genes may be effective predictors of poor prognosis in patients with glioma.

## Discussion

Due to the complex pathogenesis of gliomas, there is no specific treatment for high grade gliomas with rapid development and easy infiltration into the brain parenchyma. Among all brain tumors, high-grade glioma has the worst prognosis. Even after surgery plus radiotherapy and chemotherapy, median survival time is only 12-15 months (18). The World Health Organization revealed that the survival rate of grade IV malignant glioma is usually less than 20 months after diagnosis (19). Liu et al. improved the survival time of glioma patients by treatment with bevacizumab (20), but the effect was still not good enough. However, early detection and treatment would improve prognosis in glioma patients. Therefore, it is very important to identify new biomarkers for the diagnosis of glioma.

COL6A2 is located on chromosome 21 and contributes to the COL6 protein. Many studies have shown that the expression of COL6A2 is related to congenital atrial septal defect (ASD) (21). In addition, Manu Jokela et al. found that COL6A2 mutation can lead to delayed limb girdle muscular dystrophy (22). However, Zhu hui er et al. reported that collagen family members are high-risk factors for bladder cancer, leading to the progression of bladder cancer (23), and research confirmed COL6 as a target of MYCT1 that inhibits the adhesion and migration of laryngeal cancer cells (24). Co-expression of PLOD1 and COL6A2 results in poor prognosis in glioma (25). As for squamous cell carcinoma, Ramsey et al. considered that tumor regression after TP63 resection is associated to decreased expression of extracellular matrix relative proteins such as COL6A2 and COL17A1, LAMB3 and ITGB4 (26), indicating that COL6A2 can affect tumor progression. Among the members of the collagen family, COL5A2 is correlated to the progression of breast cancer. The extracellular matrix receptor interaction signaling pathway is up regulated, including the six collagen genes COL1A1, COL1A2, COL5A2, COL6A1, COL6A2 and COL6A3 (27). Collagen family members have gradually attracted attention from researchers in tumors. The expression of COL11A1, COL5A1 and COL6A2 is related to overall survival (OS) rate in patients with high-grade serous ovarian cancer, and their increased expression may also lead to the resistance of ovarian cancer cell lines (28). However, the significance of COL6A2 expression in the prognosis of glioma remains unclear. Our research firstly identified COL6A2 as a potential prognostic biomarker in glioma.

Although it remains unclear how the expression of COL6A2 drives glioma development, Schuster et al. demonstrate that ZFAND3 acts on the nucleoprotein complex to activate gene transcription and regulate the promoters of invasion-related genes such as COL6A2, FN1, and NRCAM, resulting in GBM invasion (29). In another study, 33 signature over expressed genes including COL6A2 were identified in GBM (30), Chen et al. divided LGG into low-risk group and high-risk group, and the high-risk group with high expression of TIMP1, COL1A1, and COL6A2 had poor prognosis (31). In this study, we found that COL6A2 has prognostic significance in glioma patients through a multi-database combination, Western blot, qPCR and immunohistochemistry suggested high COL6A2 expression in glioma. Kaplan Meier analysis based on TCGA database, GEO database, CGGA database and tissue microarray showed that COL6A2 was a prognostic factor in glioma patients. Moreover, there were significant associations of COL6A2 expression with age, clinical grade, IDH mutation, chemotherapy and 1p19q co-deletion. In glioma, the World Health Organization (WHO) believes that IDH mutations account for more than 80% of grade II/III cases (32). Studies have shown that IDH mutation is related to cell metabolism, tumor biology and tumorigenesis (33). In this study, COL6A2 expression was related to IDH mutation, indicating it has certain research significance. GSEA was performed to explore the characteristics of the high COL6A2 expression group’s gene set, and COL6A2 expression was related to “AMINO_SUGAR_AND_NUCLEOTIDE_SUGAR_METABOLISM”, “PYRIMIDINE_METABOLISM”, “STARCH_AND_SUCROSE_METABOLISM”, “PURINE_METABOLISM”, “GLUTATHIONE_METABOLISM” and “GALACTOSE_METABOLISM”.

The tumor immune microenvironment has recently become popular among researchers. There is increasing evidence that innate immune cells (macrophages, neutrophils, dendritic cells, innate lymphocytes, myeloid-derived suppressor cells, and natural killer cells) as well as adaptive immune cells (T and B cells)) contributes to tumor progression when present in the tumor microenvironment (TME) (34). Song demonstrated in the study that the expression of COL6A2 is associated with the regulation of immune cell proliferation (35). In this research, CIBERSOR results showed that COL6A2 expression was positively correlated with immune cells such as M2 macrophages, CD8+ T cells, neutrophils, gamma delta T cells, activated CD4+ T cells memory, follicular helper T cells, M0 macrophages, M1 macrophages, and regulatory T cells (Tregs). In addition, COL6A2 expression was negatively correlated with activated NK cells, eosinophils, activated mast cells, monocytes, activated dendritic cells and resting memory CD4+ T cells. Taken together, these results suggested that the expression of COL6A2 could predict glioma prognosis. Besides, the TISDB database was used to explore the correlations between COL6A2 expression and lymphocytes in the GBM dataset of gliomas. We found significant correlations between COL6A2 expression and lymphocytes. COL6A2 expression was positively correlated with regulatory T cell, type 17 T helper cell, type 1 T helper cell, myeloid derived suppressor cell (MDSC), mast cell, macrophage, monocyte, activated dendritic cell and gamma delta T cell (Tgd) amounts. Importantly, COL6A2 expression was positively correlated with immunomodulator genes. Regarding immunoinhibitory genes, COL6A2 expression had a positive or negative correlation with IL10, CD160, KDR and other genes in GBM, in addition, COL6A2 expression has a positive or negative correlation with CD28, CXCR4, IL2RA and other immunostimulatory genes in GBM. These results suggested that COL6A2 may play a very important role in the tumor microenvironment, and may participate in the development of glioma. The field of immunomodulation and immunotherapy continues to develop, and a variety of pharmacological agents have been discovered (36).

In this study, COL6A2 was associated with immunomodulatory genes. The roles played by the immunomodulatory genes related to COL6A2 expression in glioma are unknown. We examined the prognostic value of immunomodulatory genes. The prognostic model designed in the present study was composed of 16 COL6A2-related immunostimulatory genes (CD274, IDO1, IL10, KDR, CD40, CD70, CD276, CXCL1, IL2RA, IL6R, TMEM173, TNFRSF13C, TNFRSF14, TNFSF13, TNFSF14 and ULBP1). The above results showed that the risk model comprising the 16 COL6A2-related immunostimulatory genes had superior prognostic value in glioma. Extensive research has assessed immune checkpoint inhibitors in glioblastoma, and multiple studies have shown that PD-L1 is highly expressed in glioblastoma cells (37). Further investigation demonstrated that IDO1 is highly expressed in multiple types of human cancer (38). Meanwhile, IL10 significantly enhances glioma cell growth and invasion (39). These genes were associated with poor glioma prognosis in the current study. Obviously, COL6A2 is associated with these immunomodulatory genes and promotes the malignant progression of glioma.

## Conclusion

To sum up, this study demonstrated that COL6A2 expression is higher in glioma compared with normal tissues. COL6A2 may be used as a biomarker to predict the prognosis of glioma with poor survival. However, further experiments and clinical trials are essential to clarify the therapeutic value of COL6A2 in glioma.

## Data availability statement

The original contributions presented in the study are included in the article/**Supplementary Material**. Further inquiries can be directed to the corresponding author.

## Author contributions

JZ and QL carried out the studies, participated in collecting data, and drafted the manuscript. HZ and YR performed the statistical analysis and participated in its design. TJ provided help and advice on administration. JZ, QL, HZ, YR and TJ wrote the manuscript. All authors contributed to editorial changes in the manuscript. All authors contributed to the article and approved the submitted version.

## Funding

This study was funded by the National Natural Science Foundation of China (81972336).

## Conflict of interest

The authors declare that the research was conducted in the absence of any commercial or financial relationships that could be construed as a potential conflict of interest.

## Publisher’s note

All claims expressed in this article are solely those of the authors and do not necessarily represent those of their affiliated organizations, or those of the publisher, the editors and the reviewers. Any product that may be evaluated in this article, or claim that may be made by its manufacturer, is not guaranteed or endorsed by the publisher.

## Supplementary material

Supplementary Figure 1 Expression of COL6A2 in glioma. **(A)** COL6A2 was significantly expressed in TCGA-GBM than in TCGA-LGG, **(B)** The expression of COL6A2 in tissue microarray, WHO IV is significantly different from other grades, **(C)** CCLE database shows the expression of COL6A2 in different glioma cell lines.

Supplementary Figure 2 Correlation of COL6A2 expression in different immune cells in TIMER2.0 database.

Supplementary Figure 3 Tumor model establishment in nude mice and co-expression of COL6A2 and CD4, **(A)** Interfering with COL6A2 showed to significantly inhibit the growth of glioma, **(B)** Immunohistochemical assay showed that COL6A2 was co-expressed with immune gene CD4 at different concentrations. I:1:250. II:1:500.
