# Supplementary figures and images for: The pro-invasive factor COL6A2 serves as a novel prognostic marker of glioma
Source: Front Oncol. 2022 Nov 25;12:897042. doi: 10.3389/fonc.2022.897042 (PMC9732579; doi:10.3389/fonc.2022.897042)

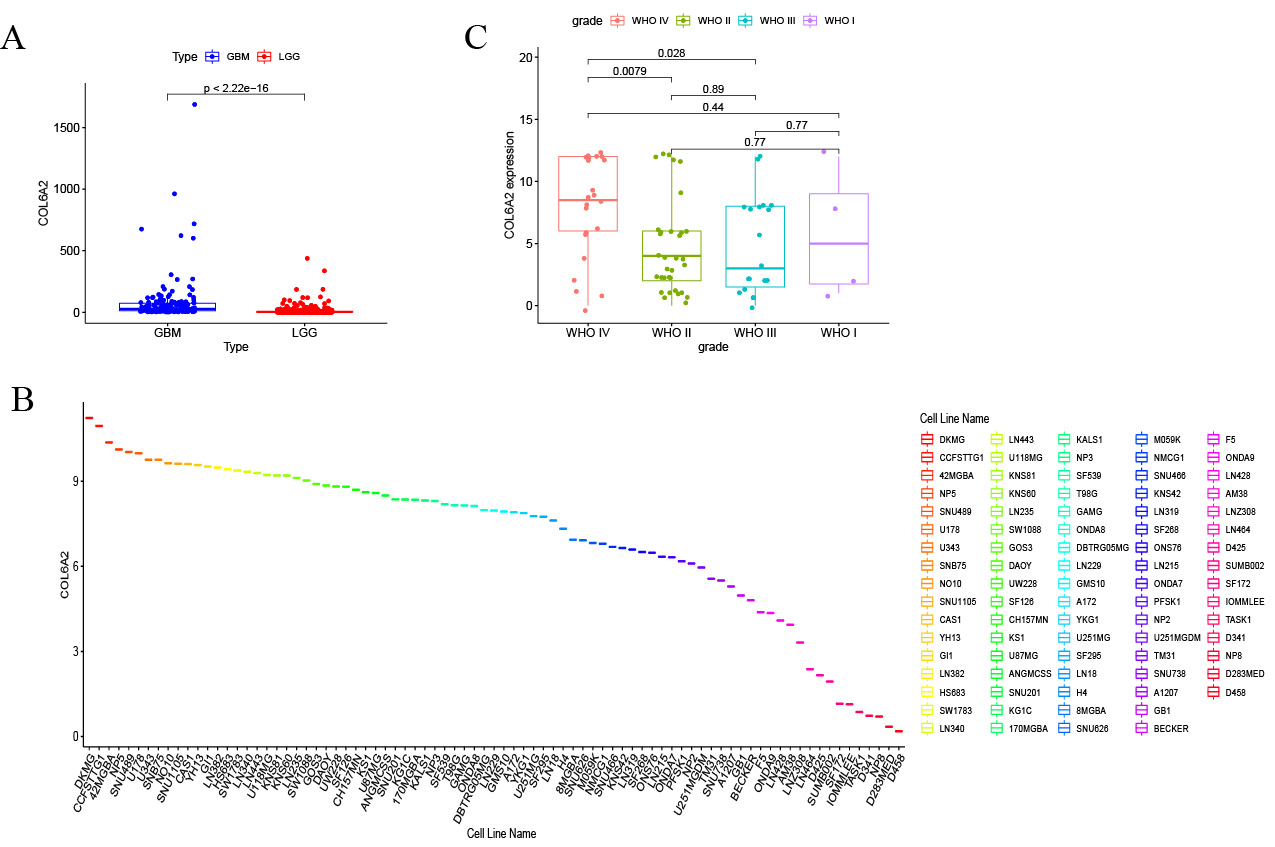

Supplement: Supplementary Figure 1 — Expression of COL6A2 in glioma. (A) COL6A2 was significantly expressed in TCGA-GBM than in TCGA-LGG, (B) The expression of COL6A2 in tissue microarray, WHO IV is significantly different from other grades, (C) CCLE database shows the expression of COL6A2 in different glioma cell lines. [file Image_1.jpeg]

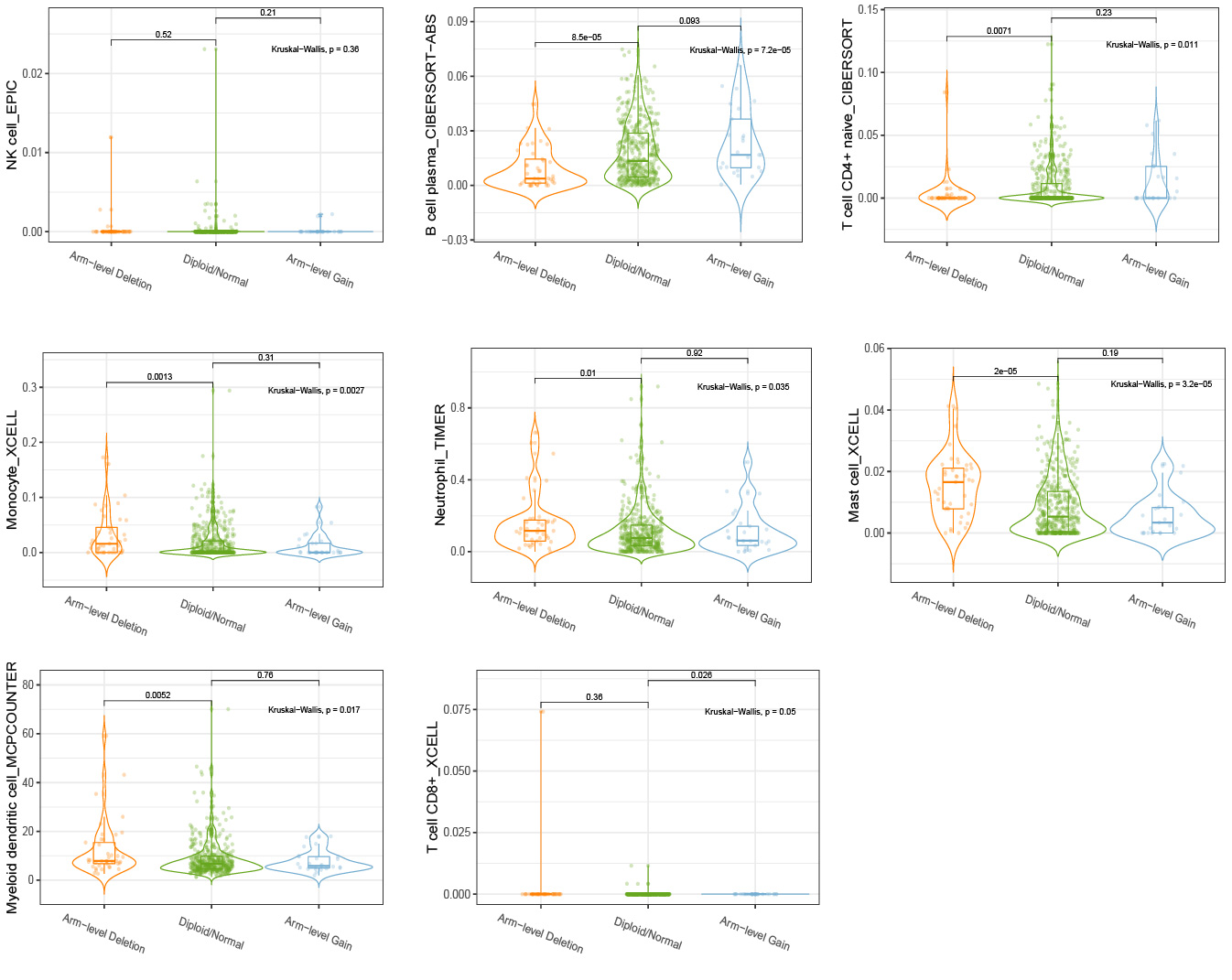

Supplement: Supplementary Figure 2 — Correlation of COL6A2 expression in different immune cells in TIMER2.0 database. [file Image_2.jpeg]

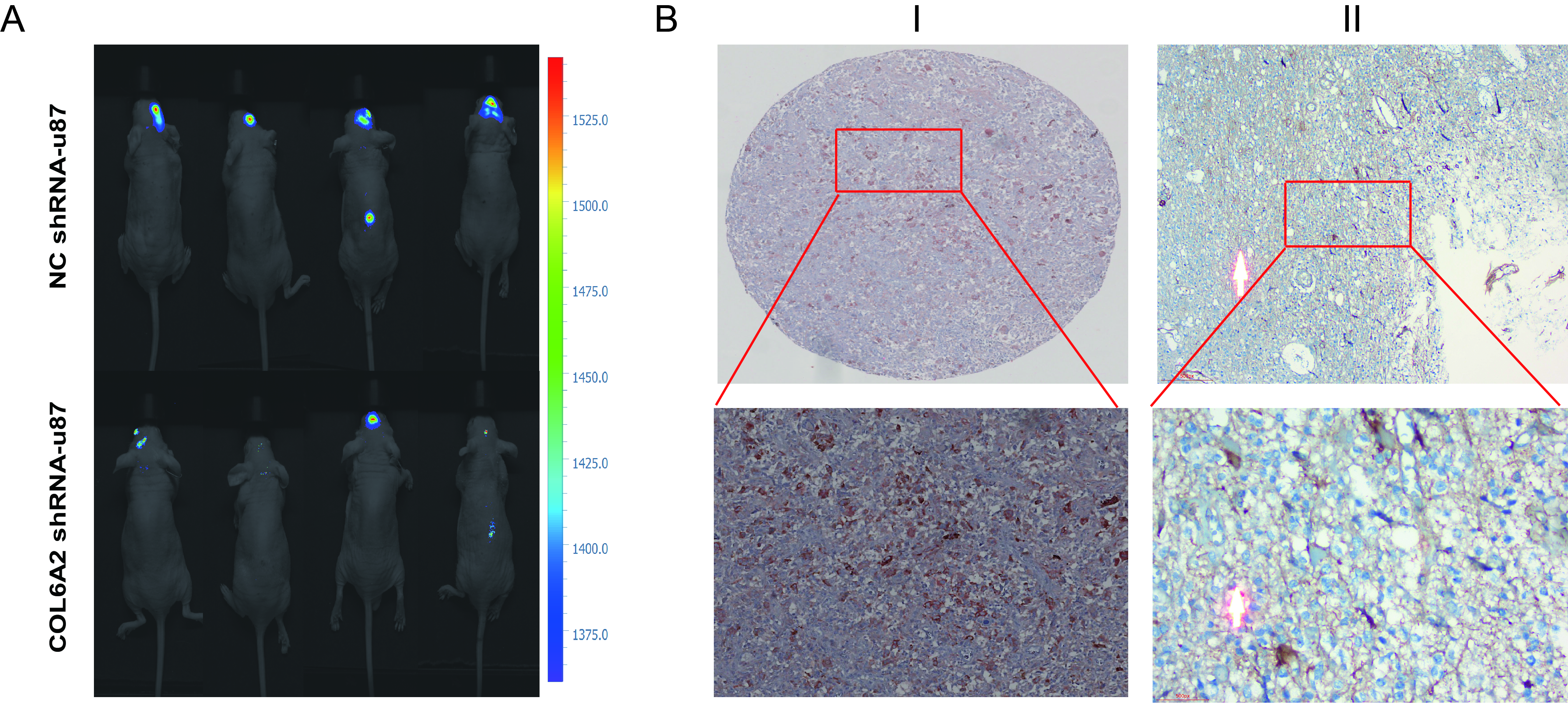

Supplement: Supplementary Figure 3 — Tumor model establishment in nude mice and co-expression of COL6A2 and CD4, (A) Interfering with COL6A2 showed to significantly inhibit the growth of glioma, (B) Immunohistochemical assay showed that COL6A2 was co-expressed with immune gene CD4 at different concentrations. I:1:250. II:1:500. [file Image_3.jpeg]
